# Epidemiological comparison between the Navarra Major Trauma Registry and the German Trauma Registry (TR-DGU®)

**DOI:** 10.1186/s13049-017-0453-2

**Published:** 2017-11-02

**Authors:** B. Ali Ali, R. Lefering, M. Fortun Moral, T. Belzunegui Otano

**Affiliations:** 1 Department of Accident and Emergency, Complejo Hospitalario de Navarra, Health Service of Navarra – Osasunbidea, Calle Monasterio de Urdax 47, 4°D, 31011 Pamplona, Navarra Spain; 2 Institute for Research in Operative Medicine (IFOM), University of Witten/Herdecke, Ostmerheimer Straße 200 (Building 38), 51109 Cologne, Germany; 3 Department of Accident and Emergency, Hospital of Tudela, Health Service of Navarra– Osasunbidea, Tudela, Spain; 4 Department of Health, Public University of Navarra, Pamplona, Spain

**Keywords:** Severe trauma, Trauma registry, Registry comparison, Quality of trauma care

## Abstract

**Background:**

International benchmarking can help identify trauma system performance issues and determine the extent to which other countries also experience these. When problems are identified, countries can look to high performers for insight into possible responses. The objective of this study was to compare the treatment and outcome of severely injured patients in Germany and Navarra, Spain.

**Methods:**

Data collected, from 2010 to 2013, in the Navarra Major Trauma Registry (NMTR) and the TraumaRegister DGU® (TR-DGU) were compared. Both registries followed the Utstein Trauma Template (European Core Dataset) for documentation of trauma patients. Adult patients (≥ 16 years) with New Injury Severity Score (NISS) being >15 points were included in this study. Patients who had been admitted to the hospital later than 24 h after the trauma, had been pronounced dead before hospital arrival, or had been injured by hanging, drowning or burns, were excluded.

Demographic data, injury data, prehospital data, hospital treatment data, time intervals, and outcome were compared. The expected mortality was calculated using the Revised Injury Severity Classification score II (RISC II).

**Results:**

A total of 646 and 43,110 patients were included in the outcome analysis from NMTR and TR-DGU, respectively. The difference between observed and expected mortality was −0.4% (standardized mortality ratio [SMR] 0.97; 95% CI 0.93–1.04) in Germany and 1.6% (SMR 1.08; 95% CI: 1.02–1.14) in Navarra. Differences in the characteristics of trauma patients and trauma systems between the regions were noted.

**Conclusion:**

The higher observed mortality in Navarra is consistent with the epidemiological characteristics of its population. However, to improve the quality of trauma care in the Navarra trauma system, certain improvements are necessary. There were less young adults with severe injuries in Navarra than in Germany. It is possible to compare data of severely injured patients from different countries if standardized registries are used.

## Background

Major trauma is a leading cause of death and disability [1]. Despite the importance of injuries, there are no strict national guidelines for trauma care in Spain, nor is there a nation-wide trauma registry. It has been shown that trauma registries are valid tools to assess and improve trauma care [2]. The great value of trauma registries lies in their potential to perform benchmarking at regional, national or international level [2].

The Navarra Major Trauma Registry (NMTR) was created in 2010 in Navarra, a region of northern Spain bordering France [3]. For benchmarking purposes, this registry follows the recommendations of the uniform Utstein style for documentation of severe trauma in Europe [4].

The outcome of emergency care of severely injured patients in Navarra has been compared previously. Gomez de Segura et al. compared the Navarra Emergency System and Atlantic Pyrenees (France) using data from 2001 to 2002. The results showed that despite more aggressive approach and employment of great resources, the French comprehensive emergency system didn’t show greater survival rates among injured patients compared to Navarra [5].

In Europe, the UK Trauma Audit and Research Network (TARN), the German Trauma Registry (TR-DGU**®**), the Dutch trauma registry, the Norwegian Trauma Registry and the Swedish Trauma Registry are well established nationwide trauma registries. The TR-DGU, a national initiative for documentation of care of severely injured patients in Germany, was founded in 1993 [6]. Nijboer et al. compared the demographics, injury mechanisms, treatment, and mortality of severely injured trauma patients (ISS >15) treated in 2005 in a level-one trauma center in Queensland (Australia) and in 59 German level-one trauma centers. The results exhibited that, despite the differences in trauma systems especially, in pre-hospital care, between both countries, the observed mortality was lower than expected in both Australia and Germany [7].

A similar study was performed by Brink et al. comparing treatment and survival of severely injured patients (NISS > 15) treated between 2006 and 2011 in Germany and Southern Finland. The authors concluded that the overall outcome results of both regions were similar and registry comparison is a feasible method of quality control in a trauma center [8].

Brilej et al. also evaluated the quality of treatment of 155 severely injured patients treated in 2006–2007 at the General Hospital Celje (Slovenia) using Trauma and Injury Severity Score (TRISS) and Revised Injury Severity Classification (RISC) methodology. The study concluded that, despite some differences between Germany and General Hospital Celje, RISC analysis performed better than TRISS in terms of discrimination, calibration and precision [9].

International benchmarking can help identify trauma system performance issues and determine the extent to which other countries also experience these. When problems are identified, countries can look to high performers for insight into possible responses. In addition, by using an international perspective, comparisons can inform benchmarks and targets for national and/or provincial governments. For successful benchmarking, meaningful performance benchmarks that can guide health policy and patient care decisions must be drawn from comprehensive, systematically collected, and valid data [10]. In Spain, data are limited at national level, and most of the well-established trauma registries are at regional or provincial level, such as the NMTR [11].

The main aim of the present study was to compare the Injury profile, treatment and outcome of severely injured patients in Navarra (Spain) and Germany using trauma registries in the respective countries.

## Methods

### Study populations

For this study, data from the NMTR and the TR-DGU® between January 1, 2010 and December 31, 2013 were used. For both registries, patients eligible for inclusion in this study were adults >15 years who had been injured by external agents with any type of intent and New Injury Severity Score (NISS) over 15 points. Patients who had been admitted to the hospital later than 24 h after the trauma, who had been declared dead before hospital arrival, who did not exhibit signs of life upon their arrival to the hospital, who did not respond to resuscitation techniques, who had been injured by hanging or drowning, or burnt patients without other traumatic injuries, were excluded.

### Trauma system in Navarra and Germany

Navarra is an autonomous province in Northern Spain with an area of 10,421 km^2^ and a population of 637,000 inhabitants. The emergency care system of Navarra is publicly funded, providing coverage to the entire population. The system is divided in three areas: Pamplona, Tudela and Estella. There are three hospitals that treat severe trauma patients in the region, through which all relevant information is included in the NMTR [3]. Navarra’s first recognized Major Trauma Service (comparable to a Level 1 trauma center), the Complejo Hospitalario de Navarra (CHN) in Pamplona, is the only tertiary referral hospital in the region. The two local hospitals (Reina Sofia in Tudela and Garcia Orcoyen in Estella) can provide initial trauma care while waiting for the right moment to transport the patient to the CHN.

Prehospital management was performed by a coordination center. The center mobilizes the resources for outpatient care (physicians or paramedics) taking into account the seriousness of the victim’s condition, referring them to the appropriate hospital emergency services. Paramedic resources (basic life support ambulances) include certified ambulance assistant technicians. Physician-staffed services (ambulances and helicopters with advanced life support) responsible for medical assistance include physicians, registered nurses and certified assistant technicians. In Pamplona, there are two physician-staffed ambulances strategically positioned that provide medical assistance to the whole area. The areas of Tudela and Estella each have one physician-staffed ambulance, at their hospitals.

Pre-hospital and hospital physicians are usually family doctors with post-graduate emergency medicine training. Around 200 trauma patients with NISS >15 are annually registered in Navarra.

Germany has a multi-payer healthcare system with two main types of health insurance: obligatory health insurance for work-related accidents and general health insurance [12]. In Germany, physician-operated emergency medical services manage most pre-hospital traumas. There are 52 physician-staffed helicopters, approximately 1000 physician-staffed ambulances and numerous paramedic-staffed ambulances. A physician at scene sees almost all serious trauma cases. Doctors working pre-hospital and hospital are physicians with a post-graduate emergency medicine training and certification; usually they are anesthesiologists [13].

In both Navarra and in Germany, trauma care is performed following the Advanced Trauma Life Support guidelines. One major difference is the resuscitation in the emergency department. In Navarra, emergency physicians perform resuscitations, whilst in Germany, it is done by a surgeon-directed trauma team. These are general surgeons with extensive experience in trauma care including fracture management [7]. Therefore, the number of involved specialties, and subsequently doctors, is often lower than in Navarra.

### The registries: NMTR and TR-DGU®

The NMTR was created in 2010 with the aim of internal and external benchmarking [3]. This is a comprehensive population registry strictly tailored to the variables and categories defined by the European Utstein Core Dataset for documentation of trauma patients [4]. Based on the Abbreviated Injury Scale (AIS), the injuries suffered by each patient are entered using a computer application. This application contains an adapted list of 152 injuries based on the revised AIS 1985 version [14], sorted by the six body regions of the Injury Severity Score (ISS), with their appropriate AIS severity level.

Database inclusion criteria were patients injured by external agents of any kind with a NISS >15. Exclusion criteria were: patients admitted to the hospital more than 24 h after injury; patients declared dead before arrival at hospital or with no signs of life on hospital arrival and no response to hospital resuscitation; asphyxia; drowning; or burnt patients with no other trauma injuries [4].

A Web application, that allows the cooperation by various users in the registry of patient data, was developed. Approximately 150 people, all doctors from the Navarra’s hospital and prehospital emergency care departments and intensive care units (ICU) of the public health system, used the application. A data manager was responsible for the general supervision and administration of the system, as well as for verifying the compliance of the inclusion criteria and of the introduction of patient data. Data was checked for completeness and plausibility; inconsistencies and missing data were handled through the hospital. Automatically generated reports on completeness of data were available at any time.

A patient can receive treatment at different hospitals: the system enables the collaboration between several hospitals and the possible management of transfers. A trauma patient may have several hospital records (one in each hospital), in which case the system generates a review by using a predefined algorithm, post-analysis of the various records. Consequently, the information on the patient’s admission status and the outcome of a trauma case are always available.

The NMTR also includes information about trauma patients who died on the scene or while being transported to the hospital [15]. Furthermore information about the severity of the injury at the scene of a motor vehicle crash, calculated by Structural Deformity Index (SDI), is also documented in the NMTR [16]. The use of the SDI can assist prehospital and hospital health care providers if particular serious injuries are suspected and anatomical and physiological criteria are not definitive.

The TraumaRegister DGU® of the German Trauma Society (*Deutsche Gesellschaft für Unfallchirurgie, DGU*) was founded in 1993. The aim of this multi-centre database is the pseudonymised and standardised documentation of severely injured patients. Injuries were coded according to the AIS, version 2008. The TR-DGU uses a reduced version with only 450 codes for documentation where similar codes with the same severity level were merged [6].

The documentation includes detailed information on demographics, injury pattern, comorbidities, pre- and in-hospital management, the course in the ICU, relevant laboratory findings including transfusions, and the outcome of every patient. The inclusion criterion is the admission to the hospital through the emergency department and subsequent ICU/ICM care or reach the hospital with vital signs and death before being admitted to the ICU.

The infrastructure for documentation, data management, and data analysis was provided by the AUC - Academy for Trauma Surgery (*AUC - Akademie der Unfallchirurgie GmbH*), a company affiliated to the German Trauma Society. The scientific leadership was provided by the Committee on Emergency Medicine, Intensive Care and Trauma Management (Sektion NIS) of the German Trauma Society. Participating hospitals submitted their pseudonymised data to a central database via a web-based application. Scientific data analysis was approved following a peer review procedure established by Sektion NIS.

The participating hospitals are primarily located in Germany (90%), but a rising number of hospitals of other countries contribute data as well (at the moment from Austria, Belgium, China, Finland, Luxembourg, Slovenia, Switzerland, The Netherlands, and the United Arab Emirates). Currently, the information of approximately 30,000 cases yearly, from over 600 hospitals, have been entered in the database. However, for the analysis in this study, only patients treated in German hospitals were considered.

The participation in the TR-DGU® is voluntary. For hospitals associated with TraumaNetzwerk DGU® however, the entry of at least a basic data set is obligatory for quality assurance.

### Comparisons

In this study, the following parameters were compared between the NMTR and the TR-DGU®: age, sex, pre-injury ASA, injury scoring, injury pattern, mechanism of injury, injury distribution, pre-hospital timings, transportation method, pre-hospital intubation, treatment at hospital, discharge destination and mortality. NMTR documents 30-day mortality defined as death within 30 days after injury or before discharge from the main hospital while TR-DGU® documents hospital mortality. For reasons of comparability patients who died beyond day 30 were considered survivors in this analysis. Regarding injury coding, in TR-DGU® injuries were graded according to reduced (450 codes) version of AIS08. This reduction was possible due to numerous detailed injury descriptions (codes) with the same severity level. Such codes were merged into a single code, conserving the appropriate severity descriptor. On the other hand, according to the revised AIS85 version, a list of 152 injuries was used in the NMTR. Note that in this list, most of injuries have the same injury severity level, for instance: grade 3 for femur fractures, etc. So, NMTR reported the injury severity level instead the full AIS code. A few injuries thus had a different severity level as in the actual version of AIS used in TR-DGU®.

**Fig. 1:**
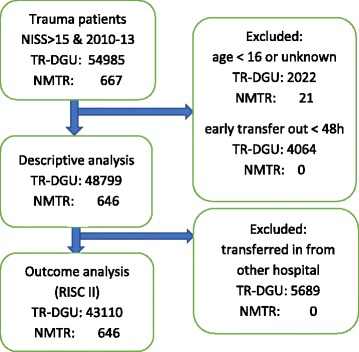
Flow diagram of included and excluded patients in the outcome analysis

Expected mortality was defined as the average value of individual prognosis derived from the Revised Injury Severity Classification II (RISC II), a prognostic score developed from the TR-DGU® data. For the TR-DGU® we excluded the early transfer out patients (< 48 h) from the descriptive analysis because there was a risk of double counting; these patients may have been documented as “transfer in” patients from the receiving hospital; furthermore, outcome was missing in these cases so they were also excluded for RISC II calculations (Fig. 1). In addition, patients who were transferred in from another hospital were also excluded because RISC II scores and initial status on admission were not available for these study subjects [6].

NMTR and TR-DGU® parameters were checked for comparability, and transformations had to be made for some of the variables before the analysis. Comparisons are based on real measurements; no imputations for patients with missing data were performed. The statistical analysis was performed using the Statistical Package for the Social Sciences (SPSS version 23, IBM Inc., Armonk NY, USA).

### Ethics approval

Navarra’s Local Medical Ethics Committee approved this study under Pyto 2016/48. The study is also in line with the publication guidelines of the TraumaRegister DGU® and registered as TR-DGU® project ID 2014–038.

## Results

### Patient characteristics

For descriptive analysis, the present study included data of 646 patients from NMTR attended in three hospitals of Navarra and data of 48,799 patients attended in 611 hospitals with documentation in TR-DGU®. Figure 1 shows the flow chart of included and excluded patients.

Patient transfer patterns were similar in both trauma systems, with major trauma patients generally transferred from smaller hospitals to major trauma centers for definitive management. In Navarra, 22.4% (170 out of 646) of the patients were transferred between facilities; while in Germany this percentage was 11.7% (5689 out of 43,110).

**Fig. 2:**
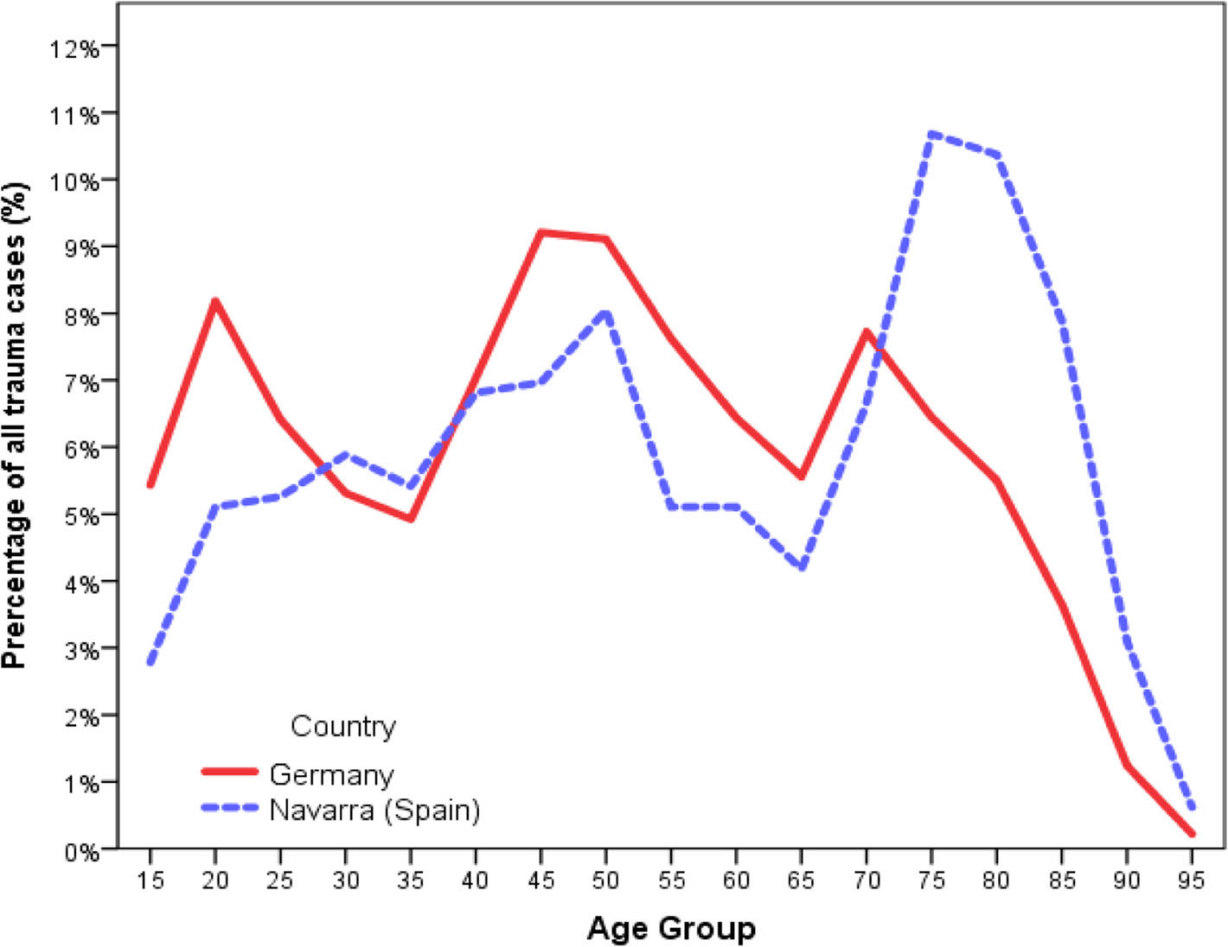
Percentage of patients by age group presenting with traumatic injuries in Navarra (Spain) and Germany

Table 1 shows the characteristics of the patients included in the analysis. The average age at the time of injury was 57.9 ± 21.9 years in Navarra and 51.6 ± 20.7 years in Germany. The percentage of trauma patients by age group between both regions is shown in Fig. 2.

**Table 1:** Characteristics of severely injured patients between Germany and Navarra (Spain)

Total no. of patients	NMTR	TR-DGU
	646	48,799
Primary cases (directly admitted from scene and treated in the receiving hospital)	476 (73.6%)	43,110 (88.3%)
Age, mean (SD)	57.9 ± 21.9	51.6 ± 20.7
	M: 58	M: 51
Male Gender	441 (68.3%)	34,919 (71.9%)
ASA (3 or 4)	55 (8.5%)	6627 (15.8%)
ISS, mean (SD) median (M)	20.4 ± 9.7	24.1 ± 11.9
	M: 17	M: 22
NISS, mean (SD) median (M)	26.5 ± 9.6	30.7 ± 13.7
	M: 25	M: 27

### Injuries: mechanism, type, and distribution

Information related to injuries is shown in Table 2. The number of traffic accidents was higher in the TR-DGU® compared to the NMTR (55.6% vs 36.3%), while more low-height falls were attended in hospitals of Navarra compared to German hospitals (34.5% vs 20.0%).

**Table 2:** Type, intention and mechanism of injury

Injury: Type, mechanism and distribution	NMTR	TR-DGU
Type of injury		
Blunt	620 (96.0%)	44,233 (95.9%)
Mechanism of injury		
Motor vehicle injury	112 (17.3%)	10,969 (23.2%)
Motorcycle injury	49 (7.6%)	6583 (13.9%)
Bicycle injury	34 (6.0%)	3699 (7.8%)
Pedestrian	36 (5.4%)	3216 (6.8%)
Gunshot wounds	3 (0.9%)	304 (0.6%)
Stabbing	11 (2.4%)	577 (1.2%)
Hit by blunt object	31 (5.5%)	1322 (2.8%)
Low fall (<3 m)	250 (34.5%)	9472 (20.0%)
High fall (>3 m)	91 (13.2%)	8275 (17.5%)
Others	29 (4.7%)	2700 (5.3%)
Road traffic accidents	231 (36.3%)	24,988 (55.6%)
Injury distribution		
Bran injury (AIS head ≥3)	397 (61.5%)	22,927 (47.0%)
Isolated head injury (AIS head ≥3, all other injuries AIS ≤ 1)	90 (13.9%)	7337 (15.0%)
Relevant thorax trauma (AIS ≥ 3)	274 (42.5%)	25,916 (53.1%)
Relevant abdominal trauma (AIS ≥ 3)	53 (8.2%)	6648 (13.6%)
Relevant injuries of the extremities (AIS ≥ 3)	77 (12.0%)	15,327 (31.4%)

**Fig. 3:**
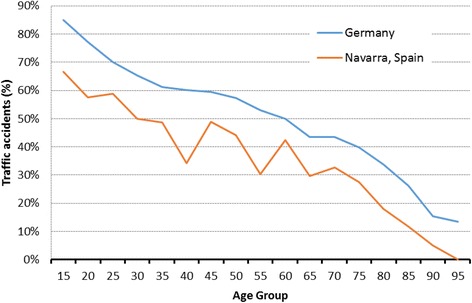
Percentage of traffic accidents by age group in both study regions

**Fig. 4:**
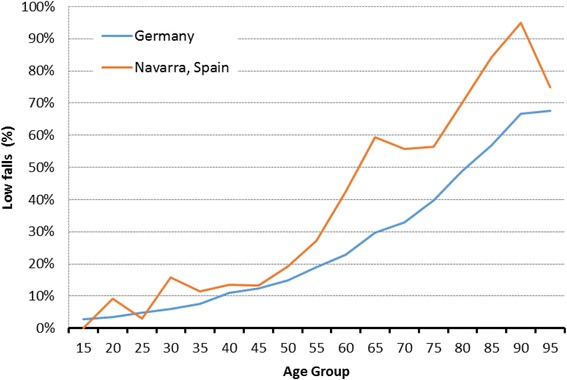
Percentage of low falls by age group in Navarra (Spain) and Germany

Figure 3 shows the distribution by age group of traffic accidents in the two registries and Fig. 4 displays the distribution by age group of low-height falls regarding to in both registries. A higher rate of chest, extremities, and abdominal trauma was determined from the Germany registry in comparison to Navarra registry (53.1% vs 42.5%, 31.4% vs 12.0 and 13.6% vs 8.2%, respectively). The prevalence of head injuries was higher in NMTR than TR-DGU® (61.5% vs 47.0%). However, isolated head injuries (e.g. AIS-code ≥3 in the head region, all other AIS-codes <2) were slightly more common in German hospitals compared to Navarra’s hospitals (15.0% vs 13.9%, respectively).

### Prehospital setting

Prehospital details between both regions are shown in Table 3. In Germany, more patients were treated by physician-staffed ambulances than in Navarra (67.4% vs. 58.1%, respectively). Helicopters were more often used to transport trauma patients in Germany (25.8%) than Navarra (4.3%). German patients receive more volume than Navarra patients (88.6% with a median of 1000 ml vs 62.3% with a median of 500 ml). Regarding response times, Navarra’s prehospital team spend more time on scene than German teams (0:34 ± 0:21 vs 0:30 ± 0:17).

**Table 3:** Prehospital data

Pre-hospital data^a^	NMTR	TR-DGU
Systolic Blood Pressure (SBP)	131.1 ± 25.0	126.1 ± 36.1
	M: 130	M: 130
Glasgow Coma Scale (GCS)	12.8 ± 3.6	11.7 ± 4.4
	M: 15	M: 14
Unconscious (GCS ≤ 8)	94 (14.6%)	9836 (24.2%)
Intubation	76 (11.8%)	15,538 (36.6%)
Cardio-pulmonary Resuscitation (CPR)	3 (0.5%)	1750 (4.1%)
Volume administration	401 (62.3%)	36,485 (88.6%)
Amount of Volume, if given (ml)	444 ± 409	1001 ± 656
	M: 500	M: 1000
Transport		
Physician-staffed ambulance	375 (58.1%)	28,197 (67.4%)
Ambulance without physician	201 (31.1%)	2324 (5.6%)
Helicopter	28 (4.3%)	10,807 (25.8%)
Private vehicle	42 (6.5%)	514 (1.2%)
Time from accident/alarm to arrival at scene	0:19 ± 0:12	0:20 ± 0:18
	M: 15	M: 15
On scene time	0:34 ± 0:21	0:30 ± 0:17
	M: 30	M: 26
Time from accident/alarm to hospital	1:06 ± 0:33	1:08 ± 0:36
	M: 1:01	M: 1:00

^a^only primary admitted cases from TR-DGU

More unconscious patients were observed in Germany (24.2%) than in Navarra (14.6%). Intubation rates were higher in Germany than in Navarra (36.6% vs 11.8%, respectively). Furthermore, patients with GCS < 9 on scene were more intubated by German prehospital teams than Navarra teams as shown in Table 4.

**Table 4:** Prehospital intubation rate according to GCS 9–15 and GCS 3–8 between Germany and Navarra (Spain)

	NMTR		TR-DGU	
	GCS 9–15	GCS 3–8	GCS 9–15	GCS 3–8
Intubation n (%)	13 (2.4%)	63 (67.0%)	5987 (19.5%)	8764 (89.2%)
No intubation n (%)	539 (97.6%)	31 (33.0%)	24,653 (80.5%)	1056 (10.5%)

### Diagnostic procedures and treatment at hospitals

Data on CT scans and surgical interventions, the prevalence in ICU, days ventilated (all intubated days and possible continuous positive airway pressure [CPAP] treatment counted together), the length of hospital stay, and the discharge destination are presented in Table 5.

**Table 5:** Hospital data and outcomes of severely injured patients of both regions

Hospital data and outcomes	NMTR	TR-DGU
Arterial Base Excess	−4.5 ± 5.0	−2.3 ± 4.9
	M: −4.0	M: −1.6
Coagulation: INR	1.2 ± 0.6	1.2 ± 0.6
	M:1.0	M: 1.1
Systolic BP	130 ± 26	126 ± 32
	M: 129	M: 130
Blood transfusion (%)	80 (12.4%)	6445 (13.3%)
Computed Tomography (CT) performed (%)	621 (96.2%)	43,158 (88.4%)
Whole body CT performed (%)	284 (44.4%)	37,260 (77.1%)
Time to first CT scan	0:43 ± 0:23	0:23 ± 0:18
	M: 0:40	M: 0:19
Time until first emergency intervention	1:52 ± 1:05	1:23 ± 0:39
	M: 1:39	M: 1:20
ICU treatment (%)	232 (36.0%)	44,621 (91.4%)
Ventilated (%)	150 (23.2%)	25,601 (52.5%)
Ventilation days (if ventilated)	5.8 ± 8.5	8.8 ± 11.5
	M: 1	M: 4
Length of hospital stay (days)	12.1 ± 14.0	21.0 ± 20.9
	M: 7	M: 16
Type of first intervention		
Damage control thoracotomy	15 (2.3%)	974 (2.0%)
Damage control laparotomy	22 (3.4%)	2304 (4.7%)
Limb revascularization	10 (1.5%)	266 (0.5%)
Interventional radiology	13 (2.0%)	116 (0.2%)
Craniotomy	39 (6.0%)	2797 (5.7%)
Observed mortality (30 days)	140 (21.6%)	6423 (14.9%)
Expected mortality (RISC II)	129 (20.0%)	6595 (15.3%)
Discharge destination (survivor only)		
Home	401 (80.8%)	21,497 (52.2%)
Rehabilitation	22 (4.4%)	13,318 (32.3%)
Another hospital	73 (14.8%)	4908 (11.9%)
Other facilities		1496 (3.6%)
Glasgow Outcome Scale at discharge		
Good recovery	416 (64.4%)	22,932 (47.0%)
Moderate disability	41 (6.3%)	11,377 (23.3%)
Severe disability	38 (5.9%)	4238 (8.7%)
Persistent vegetative state	1 (0.2%)	910 (1.9%)
Died in hospital	150 (23.2%)	7580 (15.5%)
Survivor not classified		1762 (3.6%)

More CT scans were performed in Navarra’s hospitals than in German ones (96.2% vs 88.4%, respectively). However, more whole-body CT scans were made in Germany in comparison to Navarra (77.1% vs. 44.4%, respectively). It took more time to perform the first post-admission CT scan and the first surgical intervention in Navarra versus Germany (0:43 and 1:52 vs. 0:23 and 1:23, respectively).

Patients were more likely to be admitted to the ICU in German hospitals than in Navarra’s hospitals (91.4% vs. 36.0%, respectively). High percentage of ventilated patients (52.5% vs. 23.2%), more ventilation days (8.8 ± 11.5 vs. 5.8 ± 8.5) and longer periods of hospitalization were determined in Germany in comparison to Navarra (21.0 ± 20.9 vs. 12.1 ± 14.0 days).

### Outcomes

**Fig. 5:**
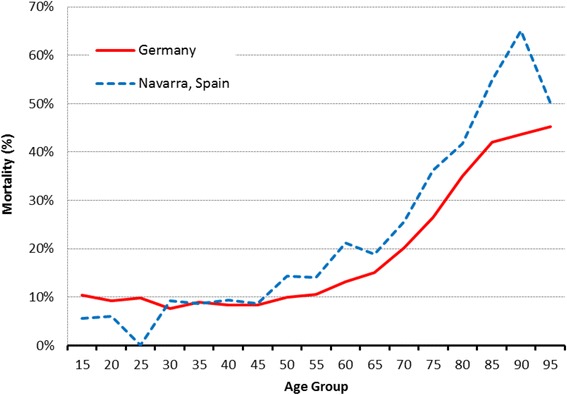
Mortality of patients by age group presenting with traumatic injuries in Navarra (Spain) and Germany

30-day mortality was 21.6% in NMTR and 14.9% in TR-DGU®. Figure 5 shows the mortality rate of trauma patients by age group in both regions. The difference between the observed and expected mortality of all patients was −0.4% (standardized mortality ratio [SMR] 0.97; 95% CI 0.93–1.04) in Germany and 1.6% (SMR 1.08; 95% CI: 1.02–1.14) in Navarra.

In Navarra, 64.4% (401 out of 646) of patients were discharged home directly from the hospital, compared with 52.2% (21,497 out of 48,799) in Germany. The number of patients discharged to rehabilitation services was higher in Germany than in Navarra (32.3% vs. 2.9%).

## Discussion

The overall results of this study show that the adjusted mortality rates of severely injured patients treated in Navarra and Germany are comparable. RISC II prognosis considered in this study display slightly lower predicted mortality than the actual mortality available from the NMTR. This might be because a score derived prognosis refers to the expected outcome in the development population [17]. For RISC II, this is a trauma population mostly treated in Germany for the 2010/11 period [6].

Other reasons that support the observed differences could therefore also might be due to the difference in the trauma care systems and/or different populations in both registries.

There are some striking differences between Germany and Navarra regarding the profile of the injured patients. Figure 2 shows three peaks for individuals aged 20 and younger, 45–50, and 70 for the German patients and two peak points for those between 45 and 50 and 75–80 for the Navarra patients. It can be presumed that occurrence of trauma between the ages of 21 to 50 is dominated by a higher number of motor vehicle accidents or work-related accidents. It can also be inferred that the increase in trauma after the age of 65 is due to the weakening of the body and reduced attention.

In addition, Lefering et al. revealed that increasing age is a risk factor for post-trauma mortality [6]. Giannadous and co-workers reported that the mortality rate for patients ≥65 years in England and Wales, in 2008, was significantly higher than in younger trauma patients [18]. In the present study, Navarra patients were older than German ones which may explain the higher mortality found in this study (Fig. 5).

Most mechanisms of injury in both data registries were classified as blunt trauma, particularly in vehicle-related accidents and falls. However, more vehicle-related accidents and a high percentage of young injured patients were seen in the German data (Fig. 3). Drunk driving, drowsy driving, and careless driving are several examples of the causes of motor vehicle accidents, and all of them are prominent in young men in general [19]. The high percentage of young injured people in Germany compared to the observed in Navarra percentage may be due to the traffic culture and the relatively liberal speed limits on the German highways. In Navarra, the speed limit in highways is 100 km/h and 120 km/h in motorways.

Both registries revealed a high rate of falls from a low height, particularly in subjects >60 years of age (Fig. 4). Older people make up a large and increasing percentage of the population. As people grow older, there is a higher risk of falls and consequent injuries. Several studies have reported high rates of fall-related mortality among the elderly [20, 21].

The proportion of elderly people continues its upward trend. Consequently, there is an increase of falls from a low height as well as of injuries such as severe head trauma, In this study, a higher number of older patients and percentage of head traumas in the NMTR have been documented in comparison to TR-DGU®.

After having identified some differences between the two trauma populations, the next step is to determine if there are regional (Germany vs Navarre) distinctions in the treatment of trauma patients and the organization of trauma care.

In Navarra, paramedic resources for patient transport are used more frequently than in Germany. This is due to the Navarra’s prehospital organization. A significant percentage of patients were transferred from the villages (periphery) in Navarra. In some cases, doctors sent patients in an ambulance accompanied by a paramedic, after attending them at the scene of the accident. In other cases, doctors attended the patient at the scene of the accident then the patient was handed over to another ambulance team. Furthermore, changes in trauma patient profiles has led to modifications of the resource activation protocols by Navarra’s coordination center. For example, transfer of conscious elder patients with isolated head injury after low fall to hospitals is delivered by paramedic ambulances. In the past, these patients were also attended by physician-staffed resources but only for the transfer. Given the limited number of physician-staffed ambulances in Navarra, and cost effectiveness requirements, protocols have been updated to adjust better to trauma needs and the seriousness of the case.

Helicopters as a mean for the transport of patients are widely used in Germany [22], mainly because of the traffic congestion at highways, while they are rarely used in Navarra. One reason that may contribute to the reduced use of helicopter resources in Navarra is the limited experience in health resource mobilization of the coordinator. Bad weather conditions in Navarra also prevent their use for the transport of trauma patients. Further investigation on the coordination and management of helicopters in Navarra should be undertaken.

Prehospital intubation rates documented in TR-DGU® were more over two-fold higher than the recorded in NMTR, with even higher values in the past [23]. There are several possible explanations for this. First, patients transported by helicopter tend to be intubated more frequently before being transported because intubation during the flight is a difficult task. In this study, the use of helicopters for the transport of trauma patients by German prehospital teams was significantly higher than in Navarra. Second, relevant chest injuries detected in prehospital setting may lead doctors to intubate patients. In this study, higher rates of chest injuries were documented in TR-DGU® than in NMTR. Third, in Germany, prehospital intubation was quite common in recent years, and even GCS 15 patients were intubated in approximately 50% of polytrauma cases in the 1990s [23]. Fourth, the low percentage of intubation seen in Navarra may be because doctors preferred airway management methods different from endotracheal intubation. On the one hand, several studies have reported increased failure rates and severe complications in trauma patients who were intubated prehospitally [24]. Furthermore, airway management with other instruments like classic laryngeal mask airway, Combitube and Laryngeal Tube have proven to be useful in prehospital airway management [25].

Furthermore GCS ≤ 8 is a general indication for intubation in Germany as well as in Navarra. In this study, NMTR has shown lower intubation rates even in patients with GCS ≤ 8 (Table 4). As previously explained, the use of supraglottic airway devices may be one of the reasons for not intubating these patients in the prehospital settings. Another reason could be the presence at the scene of the accident of an emergency physician. As has been reported that about 5.6% of trauma patients from Navarra are transferred to the hospital in private vehicles. Other factors involved as the training of prehospital emergency medical services or the time taken to transfer the patients may also have contributed to these results [26]. However, additional critical evaluation is required in this subgroup of patients, since the prehospital guidelines of Navarra recommend endotracheal intubation of all patients with GCS < 9 and it is considered as one of the quality indicators of Navarra prehospital trauma organization. The increase in the number of non-intubations for patients with initial GCS ≤ 8 can be considered as a failure of the system in the prehospital organization, so that this measure should be given special attention when reorganizing prehospital care [8].

Even with similar prehospital response times between both regions, more trauma patients received volume and more volume was administrated in Germany than in Navarra. Debate continues regarding the strategy of fluid management in trauma, however aggressive crystalloid resuscitation needs to be avoided [27]. These findings should be taken into account for further improvement in the German prehospital setting.

Hospital treating severe trauma patients in Germany are divided into three categories – supraregional (I), regional (II) and local (III), according to their resources. When participating in the TraumaNetzwerk DGU®, each trauma center has to fulfil clearly defined standards for structure, process and outcome quality, as well as criteria for expertise and capacity [28]. In Navarra, as already mentioned in the Methods section, only three hospitals treat severe trauma patients in the entire area. CHN is the only tertiary referral hospital comparable to a level I trauma center since it can provide total care for every aspect of injury – from prevention through rehabilitation. However, it does not meet the minimum requirement for annual volume of severely injured patients established by the American College of Surgeon [29]. Furthermore, no specific requirements have been established for hospitals to treat severe trauma patients in Navarra. The other two hospitals in Navarra provide primary life-saving trauma care to trauma patients as local German trauma centers (level III), especially when primary transportation to regional trauma center is not possible [28].

More CT scans were performed in Navarra in comparison to German hospitals; however, the percentage of whole body CT scans was lower in Navarra. This is explained because in our hospitals doctors are still using selective CT scan rather than whole body CT scan. There is a lack of solid scientific evidence in favor of whole body CT scan [30, 31]. Several retrospective and prospective studies agreed on a time benefit in favor of whole body CT scanning but no consensus was obtained regarding a possible survival benefit [30, 32–34]. Furthermore, despite the favorable characteristics of CT scanning, it is still associated with a high radiation dose and might affect health care costs [35]. Despite the lack of proper scientific evidence, an increasing number of trauma centers are using whole body CT scan during trauma survey, either as a supplement to or as a replacement for conventional imaging [30, 32]. It has been shown that whole body CT in high-energy trauma does not affect patient care if the patient is mentally alert, not intoxicated and does not shows signs of other than minor injuries when evaluated by a trauma-team. The risk of missing important traumatic findings in these patients is very low. Observation of the patient with reexamination instead of imaging may be considered in this group of often young patients where radiation dose is an issue [36].

It took more time to perform the first CT scan in Navarra than in Germany. Accordingly, the time to first surgical intervention also increased. Probably, doctors attending trauma patients in Navarra take more time to evaluate these patients. Furthermore, the CT scanner in Navarra hospitals is located far from the resuscitation room and it takes some time to get there and perform the imaging. It was shown that the location of the CT scanner in or near the trauma room, as opposed its location at the Radiology Department, could also have a beneficial effect on the outcome [37]. Changes should be done in hospital protocols and infrastructures to reduce these times in Navarra.

Increased ICU utilization in Germany is reflected by the high proportion of patients admitted to the ICU, as seen in this study. It can be presumed that more severe cases tend to be admitted in the ICU. In this study, the severity of German trauma patients measured by ISS and NISS, was higher than that of Navarra trauma patients. Furthermore, different indications for critical care admission may also explain the difference of ICU admission found in this study. For example, in Germany non-intubated patients with bilateral lung contusions and chest tubes are usually monitored in the ICU [38]. In Navarra, the same patient is usually monitored on the emergency observation room (discharge within 24-72 h after injury) or on a regular ward.

In Germany, a higher number of trauma patients were on mechanical ventilation as well as for longer periods. Parenchymal lung injuries, such as pulmonary contusion, may require oxygenation and ventilation support through mechanical ventilation strategies [39]. For example, in this study higher rates of chest injuries were recorded in TR-DGU® in comparison to the NMTR. Furthermore, mechanical ventilation is one of the main reasons to admit patients to the ICU [40] even in Germany [41]. In Navarra, it is often provided in the emergency observation room or general chest ward rather than in the ICU.

Study patients in Germany stayed longer in the hospital in comparison with the stay in Navarra. Some studies have examined the length of hospitalization in trauma patients, indicating that prehospital interventions such as endotracheal intubation and other procedures performed by prehospital teams at the site of the trauma can be associated with other complications such as pneumonia. This may prolong hospital stays [42]. In this study, more prehospital intubations were performed by German prehospital teams than by Navarra teams.

German patients were transferred more frequently to rehabilitation facilities. Rehabilitation services are limited in Navarra and it is often done at home or in a local hospital, while German patients are transferred to a rehabilitation center [7].

The Navarra registry and TR-DGU®, both have different data collection procedures and inclusion criteria, a limitation of this study. In Navarra, as mentioned in the Methods section, patients were included into the database thanks to the collaboration of various users from different levels. A supervisor was responsible for data completeness, made sure the inclusion and exclusion criteria were met, and of the compliance of the Utstein style variables of each registered patient. TR-DGU® contains data from many different hospitals coded by multiple people. Although multiple plausibility controls are implemented, there is no data verification source for preventing entry errors. To minimize bias due to the previously mention limitation, definitions were carefully checked, and data were transformed into comparable variables where necessary. Some variables like ventilation days (if ventilated) was defined again for this analysis, as the Utstein template is not clear [4]. The different AIS versions used by both registries is a major limitation of this study and it may have affected the results of this comparison. Specifically, the outcome prediction may be affected in this comparison and requires a careful interpretation. Although for the majority of injuries the severity levels were not changed during the AIS revisions. It has been shown that different AIS versions (e.g. AIS98 vs. AIS08) are not always comparable [43]. However, a systematic assessment of AIS85 versus AIS08 was lacking. It has previously been shown that a comparison of survival for trauma registries that use different AIS editions is possible [44]. For trauma registries, a more contemporary AIS version should be adopted in order to enhance comparability with other registries. NMTR will update its AIS severity levels according to the recommendations by the Utstein trauma template [4].

Differences across the trauma systems and hospitals offer an opportunity to compare the different ways of treating trauma patients, which would not be possible within an existing system. In this study, the analysis of large versus small trauma centers between both regions could not be carried out. On one hand, choosing only larger trauma centers would result in a biased selection of cases. On the other hand, the number of hospitals in Navarra were too small to justify a subgroup analysis regarding size of hospital.

This comparison between the NMTR and the TR-DGU® shows there are areas in need of further improvement in both systems. Actions like massive publicity campaigns, tightening the penal code and speed limits (particularly on highways), may reduce the vehicle accidents in Germany and consequently reduce the percentage of injuries among the youngest population. Changes at hospital and prehospital level are needed in both systems to improve trauma quality care in both countries. Strategies to reduce the rate and severity of low-height falls may translate into positive results for trauma patient survival rates.

## Conclusions

Both trauma registries, the NMTR and the TR-DGU®, provide data for epidemiological comparison and international benchmarking. The higher observed mortality determined in Navarra follows the epidemiological characteristics of its population. However, improvements are necessary at prehospital and hospital level to increase trauma quality care in Navarra. There were less young adults with severe injuries in Navarra than in Germany. It is possible to compare severely injured patients from different countries if standardized registries were used.
